# Supplementary figures and images for: Applying a GAN-based classifier to improve transcriptome-based prognostication in breast cancer
Source: PLoS Comput Biol. 2023 Apr 3;19(4):e1011035. doi: 10.1371/journal.pcbi.1011035 (PMC10101642; doi:10.1371/journal.pcbi.1011035)

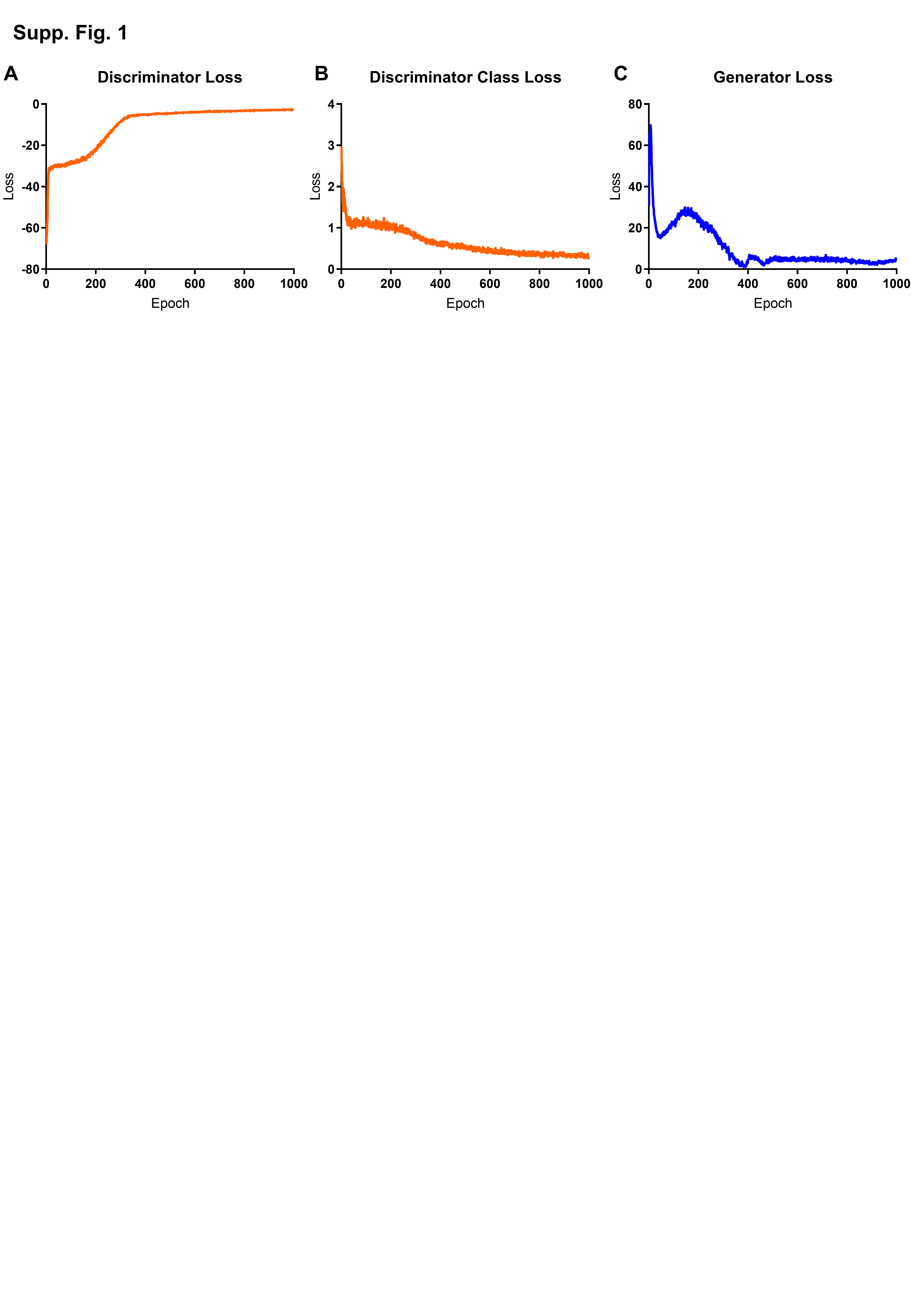

Supplement: S1 Fig — (A) Loss functions of the discriminator identifying real vs fake patients and (B) risk category. (C) Loss function of the generator. Loss functions were computed over 1000 training epochs. (TIF) [file pcbi.1011035.s001.TIF]

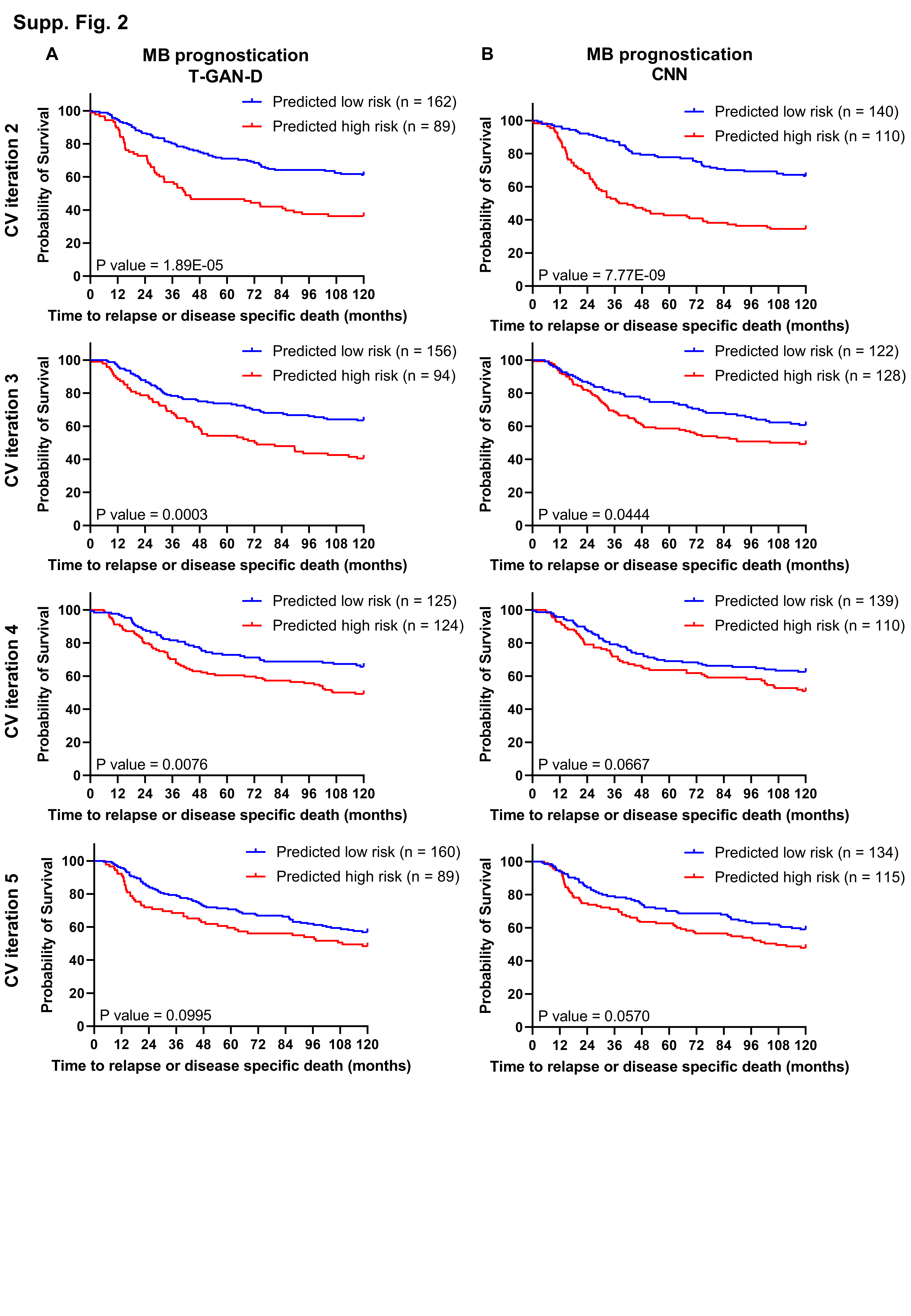

Supplement: S2 Fig — The prototyping MB cohort with all available transcriptomic data was used to compare the patient stratification obtained with the (A) T-GAN-D and (B) a classic CNN. (TIF) [file pcbi.1011035.s002.TIF]

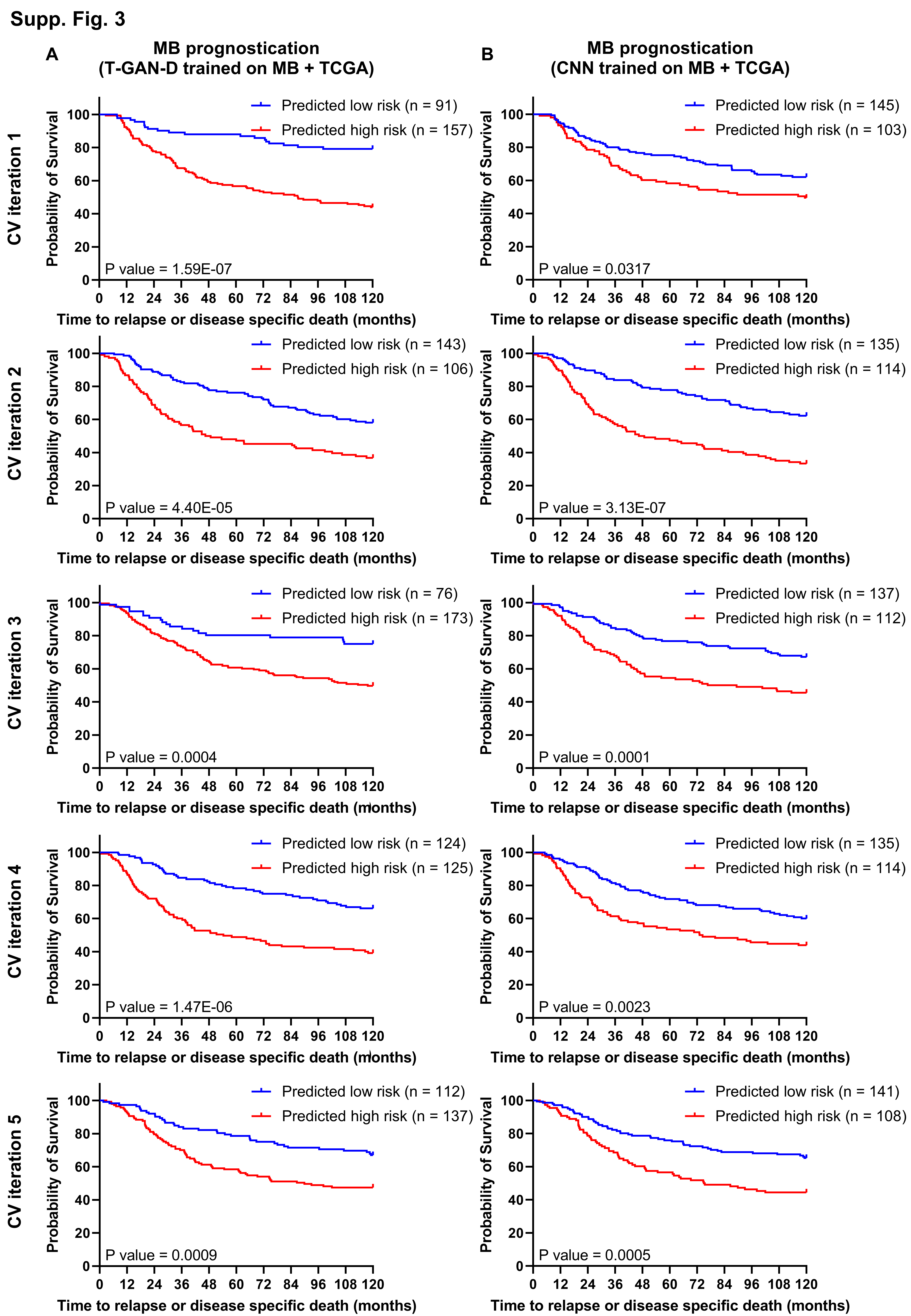

Supplement: S3 Fig — A fraction of the MB and the full TCGA cohorts were integrated to train (A) the T-GAN-D and (B) the CNN. After rescaling both datasets and filtering out the genes not available in both cohorts the risk class of the MB patients was predicted. (TIF) [file pcbi.1011035.s003.TIF]

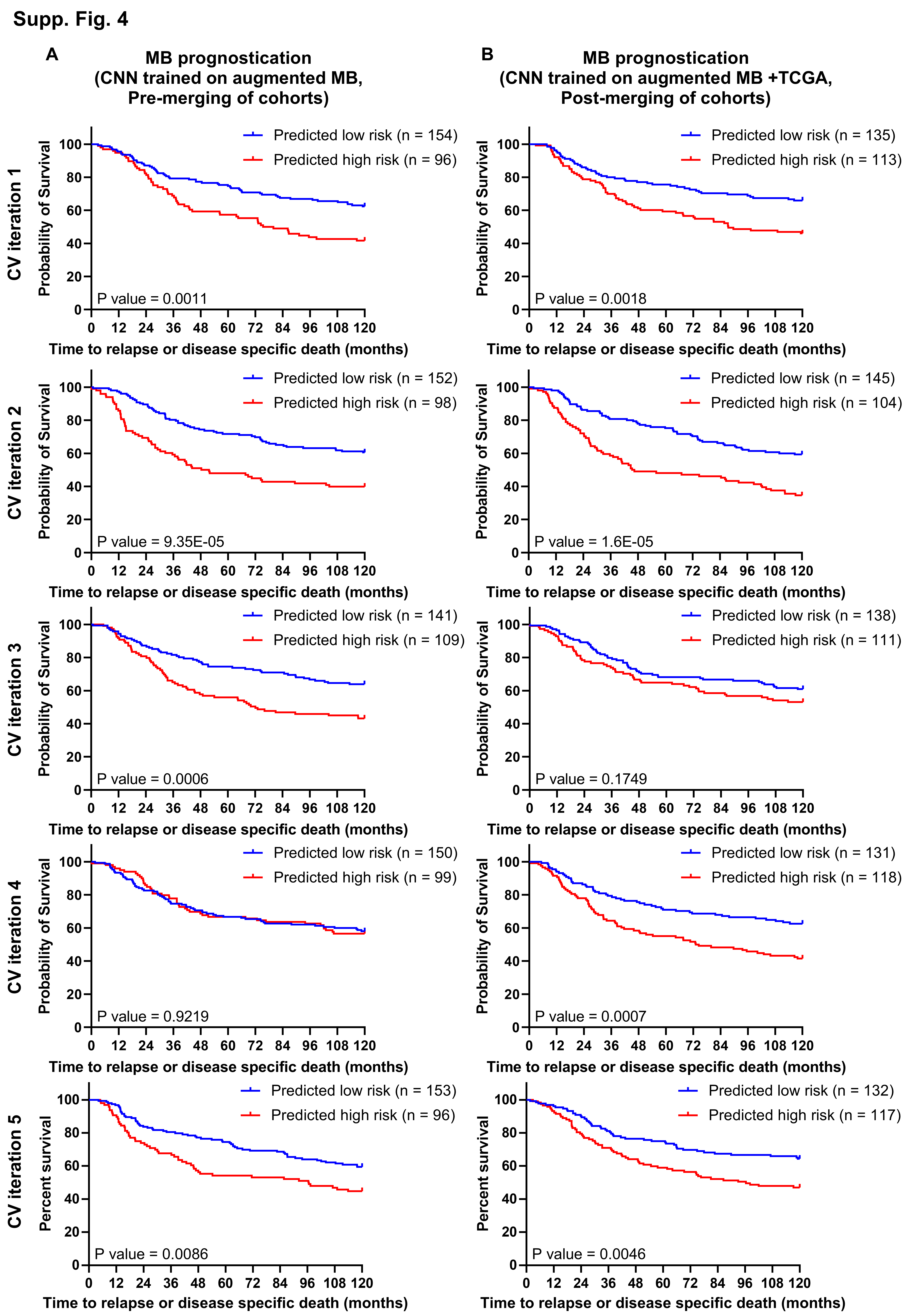

Supplement: S4 Fig — (A) Synthetic samples were generated exposing the GAN to n = 4/5 of the MB dataset. The CNN was then trained using the original training set plus n*2 synthetic samples. The remaining 1/5 samples were used as a separate test set for the CNN. The process was iterated five times and the resulting survival curves are shown. (B) Following the merging of the MB and the TCGA cohort, the GAN was exposed to 4/5 of the MB dataset and the entire TCGA dataset to produce n × 2 synthetic samples. The augmented training set was used to train a CNN and predict the risk category of the remaining unseen 1/5 MB patients. Survival curves were generated according to the predicted class. (TIF) [file pcbi.1011035.s004.TIF]

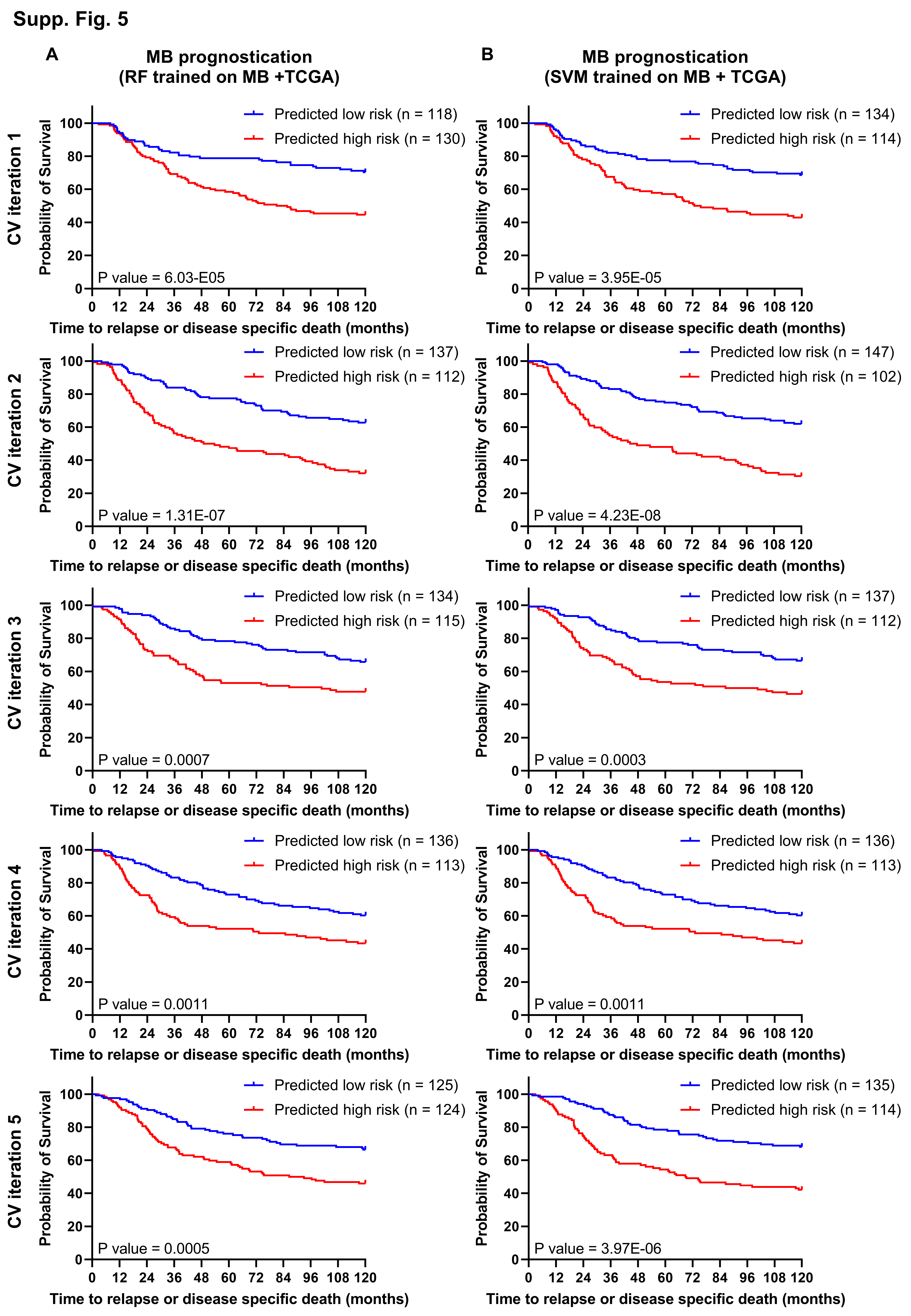

Supplement: S5 Fig — Kaplan-Meier curves generated according to the risk class predicted by (A) RF and (B) SVM at each of the 5 CV iteration. Both models were trained with data from the merged MB + TCGA cohorts to predict the risk category of MB patients. At each iteration, a feature selection preprocessing step was performed on the training set. The following number of features was selected at each iteration: CViteration 1 = 32; CViteration 2 = 33; CViteration 3 = 33; CViteration 4 = 40; CViteration 5 = 40. (TIF) [file pcbi.1011035.s005.TIF]

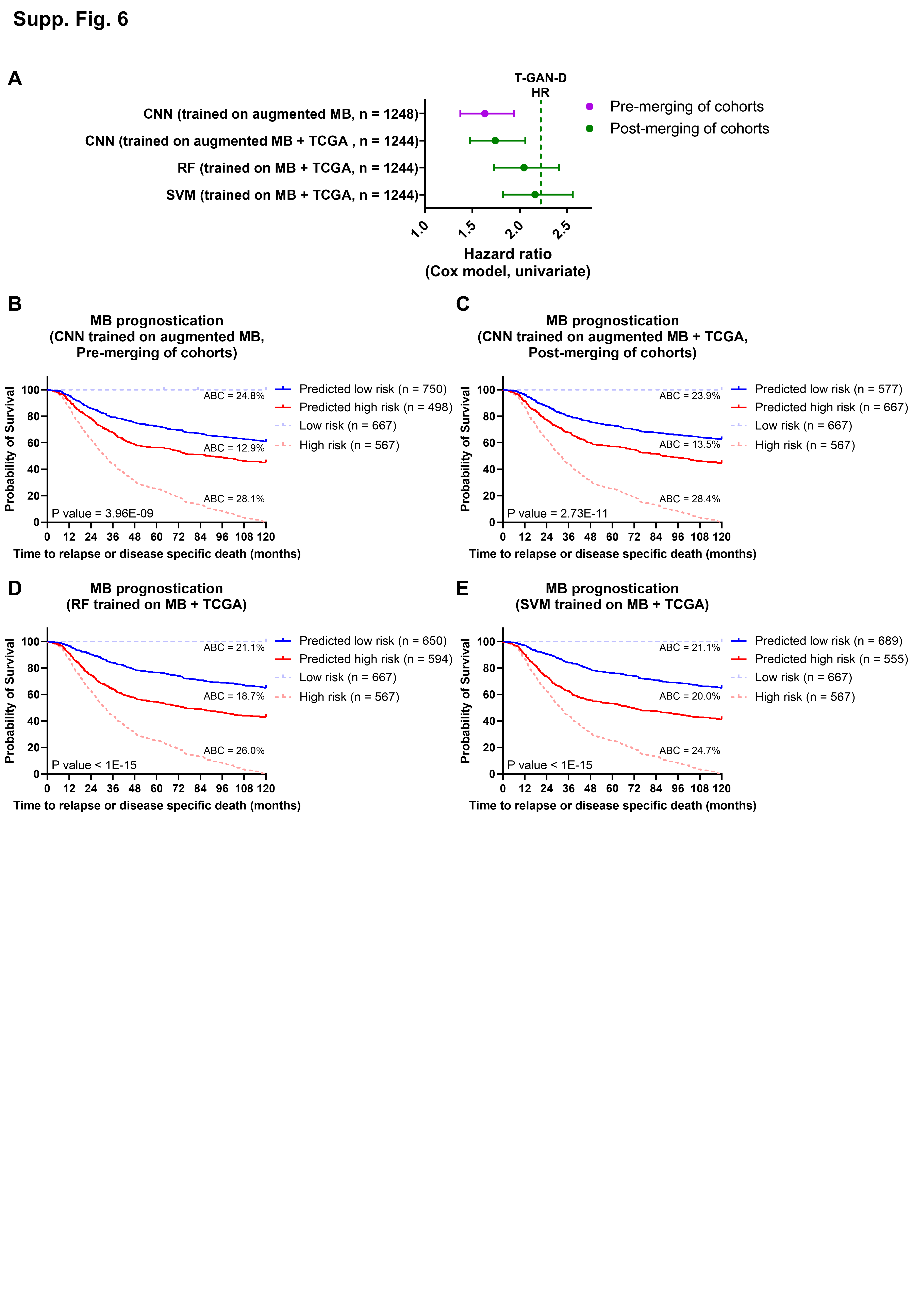

Supplement: S6 Fig — (A) Comparison of the hazard ratios (Cox model, univariate) of the CNN trained with augmented data before and after cohort merging and of RF and SVM trained after cohort merging. The dashed line represents the hazard ratio of the T-GAN-D trained after cohort merging. (B) Kaplan-Meier curves based on the pooled predictions of the CNN trained with real and synthetic samples before and (C) after merging of the cohorts. (D) Kaplan-Meier curves based on the pooled predictions of RF and (E) SVM trained after merging of the cohorts. The area between the curves (ABC) between Low risk (blue dashed line) and Predicted low risk (solid blue line), Predicted low risk and Predicted high risk (solid red line), Predicted high risk and high risk groups (dashed red line) are shown top to bottom in B and C. (TIF) [file pcbi.1011035.s006.TIF]

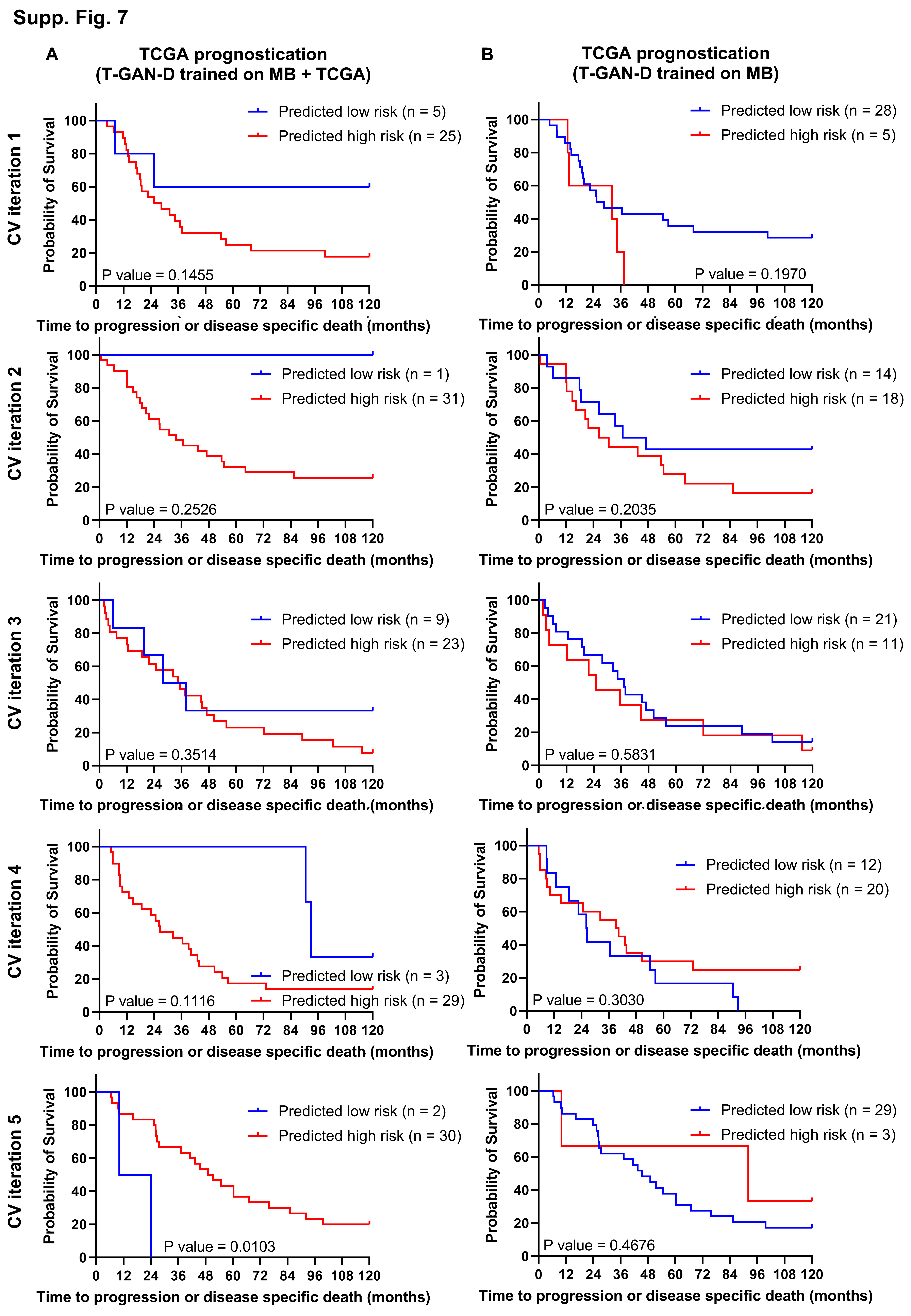

Supplement: S7 Fig — The T-GAN-D was trained (A) on the merged dataset and (B) on the MB dataset alone. After rescaling both datasets and filtering out the genes not available in both cohorts the risk class of the TCGA patients was predicted. (TIF) [file pcbi.1011035.s007.TIF]
