# Supplementary material for: Applying a GAN-based classifier to improve transcriptome-based prognostication in breast cancer
Source: PLoS Comput Biol. 2023 Apr 3;19(4):e1011035. doi: 10.1371/journal.pcbi.1011035 (PMC10101642; doi:10.1371/journal.pcbi.1011035)
Supplement: Attachment — Submitted filename: response to the reviewers.pdf [file pcbi.1011035.s013.pdf]

Response to the Reviewers
We thank the three reviewers for their helpful and generally very positive comments.
The constructive input helped us to prepare an improved, revised version. We hope the manuscript can now be recommended for publication.

On behalf of all authors
Cristiano Guttà and Markus Rehm

Reviewer #1: *This is an interesting study in which the authors develop a predictive algorithm (T-GAN-D) that serves both as a classification and as a data augmentation method, with the goal of improving the stratification of low and high risk breast cancer patients from transcriptomic data. The proposed method is evaluated on two public gene expression datasets, namely the METABRIC and TCGA cohorts. Additionally, the predictive capabilities of the T-GAN-D are compared with the performance achieved by a CNN and several established breast cancer biomarkers. Finally, the work is reproducible, since all the material needed to replicate the results obtained in this study is publicly available to the scientific community.*

- We thank the Reviewer for this very positive assessment of our study.

1. The rationale of converting gene expression data to arrays of numeric values needs to be further justified. In contrast with an image, a gene expression sample has an unstructured nature and lacks any local information that can be exploited by convolutional layers. In this way, authors should not only compare the performance of the T-GAN-D with a CNN, but also with the performance obtained by traditional ML models (such as SVM, random forest, etc.) that use gene expression vectors (without any image transformation) as input data. To counteract the great imbalance between the number of variables (high) and the number of samples (low), authors could use traditional feature selection methods to reduce the number of variables used as input to the classical ML classifiers.

- We thank the Reviewer for voicing this important line of thought. We have addressed this aspect in the revised version of the manuscript.
- In the introduction (lines 110 - 119), we now more clearly state that we ought to test the feasibility of the conversion of full transcriptome profiles to images and to assess the stratification capability of a DL based classifier fed with such unstructured arrays of pixels. Since both the CNN and T-GAN-D were able to significantly separate low vs high risk patients, this suggests that hidden underlying features are present in the images and can be captured by the DL algorithms. In future work, we plan to study the impact of the arrangement of the features/pixels on the prognostic performance, yet this goes beyond the scope of the current manuscript. We refer to this now in the outlook (lines 431 - 439).
- As suggested by the Reviewer, we also applied RF and SVM to the combined MB + TCGA dataset to predict the risk category of MB patients. In order to ensure comparability of the results, training and test sets used in the five cross validation (CV) iterations were

coherent with those of the experiments summarized in Fig. 3. The number of variables was reduced using the *Fast Correlation Based Filter* feature selection as described in the updated methods section. An additional paragraph describing the procedure (Feature selection and application of classic machine learning algorithms) was added to the methods section. The results were displayed in two additional supplemental figures, representing the survival curves generated from each CV iteration (Supplementary Figure 5), and the Kaplan Meier curves of the pooled predictions complemented by the resulting Hazard Ratios (Supplementary Figure 6A, C-D). The performance metrics are summarized in Supplementary Table 4. The results indicate that the T-GAN-D approach performs as good if not slightly better, than RF and SVM approaches and does so without prior feature selection.

2. Accuracy is used as a metric to evaluate the classification performance of T-GAN-D and CNN. However, additional metrics, e.g., specificity, sensitivity, should also be computed to perform a thorough evaluation of the classification capabilities of the models, by evaluating their performance separately for each of the two classes (high and low risk). This is specially relevant when evaluating the models on the TCGA dataset (last subsection of Results section), where there is a considerable difference between the number of high and low risk patients.

- We considered the survival analysis, Log-rank P values and Hazard Ratios to be the primary performance indicators in our study, since the dimension of time is taken into account (e.g., the classification as low risk of a patient experiencing relapse at nine years after diagnosis, is "less wrong" than the classification of a patient as low risk experiencing relapse after one year). These nuances cannot be captured by accuracy, sensitivity or specificity, despite these metrics being commonly used to evaluate binary classifiers. We apologize should this not have been clearer and have accordingly specified this better in the text (lines 267 - 270).
- Even though often ignored in related studies, we nevertheless computed accuracies to offer this metric as an additional indicator of performance. As suggested, we now also added sensitivity and specificity to all Supplementary Tables to complement the provided information. When analyzing the TCGA dataset, the specificity expectedly varied widely depending on the composition of the run and the scarcity of low risk patients.

Minor comments:
- Line 243: "and" should be changed for "an".
- Fig. 1F: "Predicted" should be removed from the legend of the figure.

- We thank the Reviewer for highlighting this. This has now been corrected.

- In Fig. 4A, authors need to clarify the meaning of "Pre" and "Post" transcripts filtering.

- We thank the Reviewer for highlighting this potential source of confusion. Fig. 4A was modified using the nomenclature *Pre-* and *Post-merging of the cohorts*, in line with the updated definition used in the methods section *Data integration* (line 136).

Reviewer #2: *This manuscript proposed to use of a GAN-based data augmentation strategy to alleviate the overfitting of the classification model, with the aim of predicting the prognostic risk of breast cancer patients. The manuscript is in general well-written but needs to be checked thoroughly. I may have some concerns that need to be addressed.*

- We thank the Reviewer for highlighting the quality of our study. All remaining comments have been addressed in the revised version.

Can you provide the number of patients and genes in both the MB cohort and TCGA cohort before and after defining the low and high-risk categories?

- We assume that the comment refers to the definition of the inclusion criteria. The numbers of patients and transcripts of the MB (n = 1904 patients, m = 18543 transcripts) and TCGA cohorts (n = 1101 patients, m = 20532 transcripts) were added to the "*Data integration"* paragraph of the methods (lines 123 and 129). The number of patients in the respective groups after the application of the inclusion criteria is shown in the panel depicting the ground truths (Figure 1E-F) and in the methods section (lines 153 – 154)

Please explain the rationality of the category definition of high-risk and low-risk patients.

- The category definition is provided in the methods section under "Inclusion criteria and category definition". Patients experiencing relapse or disease specific death before 10 years were classified as high risk. A follow up time of 10 years is widely accepted in the field (Varnier et al., 2021. Cancers. DOI: 10.3390/cancers13194840) and used in commercially available prognostic tests together with relapse endpoints (e.g. Prosigna, Mammaprint). In particular, in order to directly compare the prognostic performance of the T-GAN-D with a widely used gene expression signature (see Fig. 4A), the definition of low and high risk patients also aligns with categories specified in Xia et al. (Xia et al., 2019. Nature Communications (DOI: 10.1038/s41467-019-13588-2). There, the authors used a research-based implementation of the commercially available *Prosigna Risk of Recurrence – Proliferation score* (ROR-P) based on the work of Parker et al. (Parker et al., 2009. Journal of Clinical Oncology. DOI: 10.1200/JCO.2008.18.1370) to assess the risk class of MB patients.

*How to convert the expression values of 18543 genes into the matrix of size 144*144?*

- We amended a typo in Fig. 2A that may have caused the Reviewer's comment. The size of the square matrix of the first layer of the generator is 18x18. The size of the matrix doubles at each step (36x36, 72x72, 144x144), resulting in an array of 20736 pixels in

total (see updated Fig. 2A). The values missing to complete the final square matrix were filled with random values sampled from a -1 to 1 uniform distribution. We updated the methods section "*GAN architecture*" accordingly including details on the initial matrix size (lines 178 - 184)

What is the difference in time complexity between T-GAN-D and CNN?

- In terms of trainable parameters, the T-GAN-D is approximately 2.75 times more complex than the CNN. When dealing with small and/or unbalanced datasets, CNNs however may suffer from under or overfitting (Nandhini Abirami et al. 2021, Complexity. DOI: 10.1155/2021/5541134). Therefore, in our use case, we traded a less computationally expensive network (CNN) with a more complex but stable model implementation (T-GAN-D).

  GAN:
  Discriminator:
  Total params: 450,054
  Trainable params: 224,899
  Non-trainable params: 225,155

  Generator:
  Total params: 462,384
  Trainable params: 237,229
  Non-trainable params: 225,155

  CNN:
  Total params: 167,682
  Trainable params: 167,490
  Non-trainable params: 192

It shows 161 patients are predicted low-risk group and high-risk group in both Fig.5 (B and C). I think the experiments resulted from the TCGA-BRCA dataset instead of MB + TCGA, or MB.

- Indeed, in Fig. 5B the network is trained using both cohorts (MB + TCGA) while in Fig. 5C the training is performed with the MB cohort alone. In both settings the test set used was the TCGA. We apologize if it was not clear enough from the figure labeling that T-GAN-D was used to prognosticate the TCGA cohort. To assist readability, we modified the titles of Fig. 5B-C and made similar amendments to all the KM graphs displayed in this study.

Current methods under comparison do not provide a conclusive benchmark.

- Without additional information, if remains unclear to us what exactly the Reviewer would consider a conclusive benchmark. Since our main goal was to benchmark the T-GAN-D with prognostic methods currently used in a clinical context, several biomarkers routinely tested (Harbek et al., 2019. Nature Reviews Disease Primers. DOI: 10.1038/s41572-019-0111-2) were compared in Fig. 4A (i.e. Estrogen Receptor expression, Human Epidermal Growth Factor receptor 2 expression and Progesterone Receptor expression). In addition, the stratification performance of our classifier was compared to a research-based implementation of the commercially available *Prosigna Risk of Recurrence – Proliferation score.* This second generation gene expression signature is commonly integrated in the clinical decision process, therefore was chosen as a suitable benchmark for our classifier. From a computational, rather than clinical, point of view, Reviewer #1 suggested the comparison of T-GAN-D with classic ML algorithms (RF and SVM). These results are now displayed in Supplementary Fig. 5 and 6 and Supplementary table 4. We hope that overall this sufficiently addresses this comment.

Many descriptions are not standardized. such as inconsistent words (BRCA-TCGA and TCGA-BRCA).

- We thank the reviewer for highlighting the inconsistency. The manuscript was thoroughly checked and amended accordingly.

Reviewer #3: *The paper shows the application of a generative adversarial network (GAN) to improve prognosis in breast cancer using deep learning (DL). Specifically, the authors use transcriptomic data to identify low-risk and high-risk breast cancer patients. They process the gene expression data to turn it into images that can be more easily used by two DL models: a convolutional network (CNN) and an Auxiliary Classifier Wassertein GAN with gradient penalty (AC-WGAN-GP). The METABRIC and TCGA-BRCA selected cohorts are two of the largest and best-annotated breast cancer databases, an excellent choice for testing the efficacy of models.*

- We wish to thank the reviewer for highlighting the strength of the use case chosen for our study.

The paper shows that the discriminator of the AC-WGAN-GP model trained with the real processed images and the synthetic images created by the generator, as is logical in the normal process of a GAN, can be used as a DL classifier model to identify the patient prognosis. This discriminator, named by the authors as T-WGAN-D, improves the results obtained by a CNN. It should be noted that the paper does not present a new model or data augmentation (DA) application, but rather an application variant of part of an AC-WGAN model (the discriminator). The paper uses a non-precise definition of DA. It is not correct to consider DA the use of synthetic images in the training of a model that precisely requires these for its training. If the generated synthetic images were also used to augment the training set of another model, such as the CNN, it could be considered DA. Beyond this critique, the application of T-WGAN-D is

novel enough considering the problem posed and it improves the performance achieved by the CNN model.

- We thank the Reviewer for this very constructive critique. Indeed, we exploit the training of the GAN itself and afterwards use its discriminator as a standalone classifier in a fashion similar to transfer learning. During the training epochs, the discriminator learns category-specific features while being exposed to the additional images produced by the generator, so that the process aided by data augmentation. The reviewer convinced us that the use of the term *data augmentation* in this context can be misleading. We therefore changed the title of the manuscript to "Applying a GAN-based classifier to improve transcriptome-based prognostication in breast cancer" and modified the overall text body following the Reviewer's advice (see lines 46, 223 – 224, 269 – 267).

I pointed out some issues that needs to be addressed:
- A paragraph that explains structure of paper can be added at the end of the introduction section.

- We feel this is a great suggestion to improve our manuscript. We have now added such a paragraph at lines 110 -119.

- Other references that also studied GAN-based data augmentation techniques on TCGA mRNA data can be added.
o Moreno-Barea, F.J., Jerez, J.M., Franco, L. (2022). GAN-Based Data Augmentation for Prediction Improvement Using Gene Expression Data in Cancer. In International Conference of Computational Science – ICCS 2022. Lecture Notes in Computer Science, vol 13352. Springer, Cham. doi: 10.1007/978-3-031-08757-8_3
o Kwon C, Park S, Ko S, Ahn J. Increasing prediction accuracy of pathogenic staging by sample augmentation with a GAN. PLoS One. 2021 Apr 27;16(4):e0250458. doi: 10.1371/journal.pone.0250458

- We thank the reviewer for the input. Both studies are now integrated into the discussion (lines 423 - 429).

The authors should compare the performance of T-WGAN-D against other traditional methods and models used to identify high-risk breast cancer patients.

- Traditionally, the status of three main markers is universally recognized as indispensable for the appropriate prognosis of breast cancer patients and are routinely tested at diagnosis: the expression of estrogen receptor, progesterone receptor and human epidermal growth factor receptor 2. (Harbek et al., 2019. Nature Reviews Disease Primers. DOI: 10.1038/s41572-019-0111-2). In addition to these comparisons, the T-GAN-D was compared to the Risk of Recurrence score, a second generation multi-gene expression signature widely used for breast cancer prognostication. Even though not

relevant in clinical practice, we now also compared the T-GAN-D with RF and SVM, as suggested by Reviewer #1 (see there).

- The authors should try data augmentation on CNN, as increased variety may lead to improved performance. DA involves using synthetic data to train models that do not use it primarily.

- Following the suggestion of the reviewer, the GAN was used to generate n*2 synthetic samples that were added to the training set of the CNN in two different settings.
- In the first experiment, the synthetic samples were generated exposing the GAN to n = 4/5 of the MB dataset. The remaining 1/5 was used as a separate test set for the CNN. The process was iterated five times and the resulting survival curves are shown in Supplementary Fig. 4A.
- In the second experiment, following the merging of the MB and the TCGA cohort, the GAN was exposed to 4/5 of the MB dataset and the entire TCGA dataset to produce synthetic samples. The augmented training set was used to train a CNN and predict the risk category of the remaining unseen 1/5 MB patients (Supplementary Fig. 4B). The KM curves generated pooling the predictions of the five cross validation iterations are displayed in Supplementary Figure 6B-C and the resulting Hazard Ratios are reported in Supplementary Figure 6A. All the evaluation metrics are reported in Supplementary Table 3.

- At line 243, the authors wrote " Introducing and independent cohort improves MB patient classification." Please review this error.
- In Figure 4.A it appears that AC-GAN was used, but T-GAN-D is mentioned in the text. Please review it.

- We thank the reviewer for highlighting these oversights. We corrected both mistakes.

- In the section on the results of the union of both cohorts to predict on one of them, the re-training of only the discriminator is mentioned. However, in Figures 3 and 5 the generator training also appears. These create confusion please revise accordingly.

- As correctly stated by the reviewer, the retraining involves both the generator and the discriminator networks. The text was revised to avoid confusion (lines 296, 340).

- It would be convenient to mention transfer learning in the section described in the previous point. The retraining procedure is transfer learning, not DA.

- We thank the reviewer for this valuable suggestion. We now mention the concept of transfer learning when explaining the reuse of the trained discriminator as a standalone classifier (see lines 113 and 265).

- Future studies can be added with possibility of employing other deep learning architectures.

- We assume that the Reviewer refers to an outlook on future work based on DL algorithms in our discussion section. We now integrated such a paragraph to address this point (lines 429 - 441).
