# Supplementary material for: Applying a GAN-based classifier to improve transcriptome-based prognostication in breast cancer
Source: PLoS Comput Biol. 2023 Apr 3;19(4):e1011035. doi: 10.1371/journal.pcbi.1011035 (PMC10101642; doi:10.1371/journal.pcbi.1011035)
Supplement: Attachment — Submitted filename: responses to the reviewers 2.3.pdf [file pcbi.1011035.s014.pdf]

Response to the Reviewers

We thank Reviewers 2 and 3 for approving the implementation of the requested revisions and recommending our revised manuscript for publication. Reviewer 1 requested some additional text linked to work requested during the initial revision period. We have now accordingly amended the manuscript to satisfy this further request.
We hope that the manuscript is now acceptable for publication.

With best wishes on behalf of all authors
Cristiano Guttà and Markus Rehm

Reviewer #1*: Overall, the authors have produced an improved revised version of the manuscript. However, some concerns must be addressed to meet the publication criteria:*

1. If we compare the results obtained by the traditional machine learning (ML) models (Supplementary Figures 5 and 6, as well as Supplementary Table 4) and the proposed T-GAN-D model, the T-GAN-D approach does not outperform the support vector machine (SVM) algorithm. Consequently, authors should justify in the manuscript the development of a complex deep learning (DL)-based methodology when a simpler and straightforward approach based on a feature selection method and a classical ML algorithm achieves an equivalent level of performance.

While the main and most important objective of our study was to test the T-GAN-D against marker panels and prognostication approaches relevant in clinical practice, we now also extended the discussion section further to satisfy this reviewer request.
Key aspects include that deep neural networks are able to perform feature extraction automatically, allowing classification without prior feature selection. This translates into more general applicable classifiers, avoiding feature selection steps that can influence classifier performance, as shown e.g. for SVM or RF applied to multi-omics dataset (Li et al., 2022. BMC Bioinformatics. https://doi.org/10.1186/s12859-022-04962-x). This is pointed out in the revised discussion now at lines 436 - 440.
Furthermore, it is assuring that our first prototype implementation of the T-GAN-D can compete with general purpose ML algorithms, even though no knowledge-driven spatial arrangement of the features in the images was performed yet (lines 450 – 454). Implementing this in future work (e.g. spatially arranging transcript pixels taking biological relations within signaling pathway or biological activities into account) is an exciting avenue to follow. Indeed, the value lying in such strategies was demonstrated in other studies (already covered in the previous version of the discussion and now further extended, see lines 444 – 450; Sharma et al., 2019. Scientific Reports. https://doi.org/10.1038/s41598-019-47765-6; Lyu et al., 2018. bioRxiv. https://doi.org/10.1101/364323; Ma et al., 2018. arXiv. https://doi.org/10.48550/arXiv.1804.05283).
Finally, our image-based pipeline lends itself to be tested on multi-omics data in the future. Instead of using a single 2D array of pixels, multiple layers of -omics data could be converted

into separate, yet matching, channels. Integrative breast cancer datasets containing -omics data (e.g. the mixOmics breast.TCGA dataset, containing matching mRNA, miRNA and proteomics data) could be used to generate such images, allowing the analysis of three (or more) separate -omics domains at once. Underlying interactions between multiple domains organized in 3D arrays cannot be captured by classic ML algorithms, which consider features individually. This has now also been added to the discussion at lines 454 - 459.
We thank the reviewer for suggesting this addition to the manuscript.

2. Authors should describe in the manuscript how sensitivity and specificity metrics were calculated, i.e. in the context of binary classification, which category (either high or low risk) was considered as positive, and which one was considered as negative.

For the calculation, we considered individuals at high risk as “positive” and individuals at low risk as “negative”. We added this information to the methods sections (lines 220-226) together with the formulas used for calculating sensitivity and specificity.

Reviewer #2*: The authors have answered my comments clearly.*

Reviewer #3*: The article shows many improvements compared to the first revised version. All the issues raised by the reviewers have been addressed and the scientific and technical quality of the article has increased with the changes introduced.*
